# Targeting dyspnoea through exercise intensity: insights from pulmonary rehabilitation and beyond, a narrative review

**DOI:** 10.3389/fresc.2026.1874766

**Published:** 2026-07-14

**Authors:** Christophe Romanet, Johan Wormser, François Philippart

**Affiliations:** Department of Intensive Care, Groupe Hospitalier Paris Saint Joseph, Paris, France

**Keywords:** dyspnoea, exercise training, intensity, pulmonary, rehabilitation

## Abstract

Dyspnoea is a severe symptom, causing immediate distress and significantly impairing health-related quality of life, particularly in chronic conditions. Pulmonary rehabilitation is a cornerstone in the management of chronic obstructive pulmonary disease, as supported by evidence highlighting its efficacy in improving dyspnoea, aerobic capacity, and health-related quality of life. In this multidisciplinary approach, much of those physical effects are enabled by a physiotherapist-led intensive training strategy: exercise training. In recent years, numerous studies have supported the benefits of exercise training as an effective intervention for several other chronic diseases (including interstitial lung disease, long-covid, obesity, neuromuscular disorders). Despite differences in pathophysiology, these populations share common mechanisms responsible for the chronicity of dyspnoea, including deconditioning and peripheral muscle dysfunction, which can be targeted by exercise training. After exploring the physiological mechanisms by which exercise training alleviates chronic dyspnoea, this state-of-the-art narrative review aimed to provide a readable practical synthesis to help both those learning about exercise training in pulmonary rehabilitation and those treating the individuals affected by chronic dyspnoea. A comprehensive search strategy was employed to identify relevant studies, with publications ranging from January 2020 to April 2025 from MEDLINE, the Cochrane Database, and ClinicalTrials.gov. We support the importance of the higher intensities of exercise training as a pivotal component in enhancing the individualised therapeutic potential of exercise training across diverse disease states. We also summarise evidence on muscle-level adaptations and special attention is given to the potential role of eccentric contractions in enhancing mechanical efficiency and reducing fatigue resistance, which could be a promising area for future research. Finally, we provide practical guidance on subjects’ selection, baseline assessment, intensity prescription and monitoring.

## Introduction

**Figure 1:**
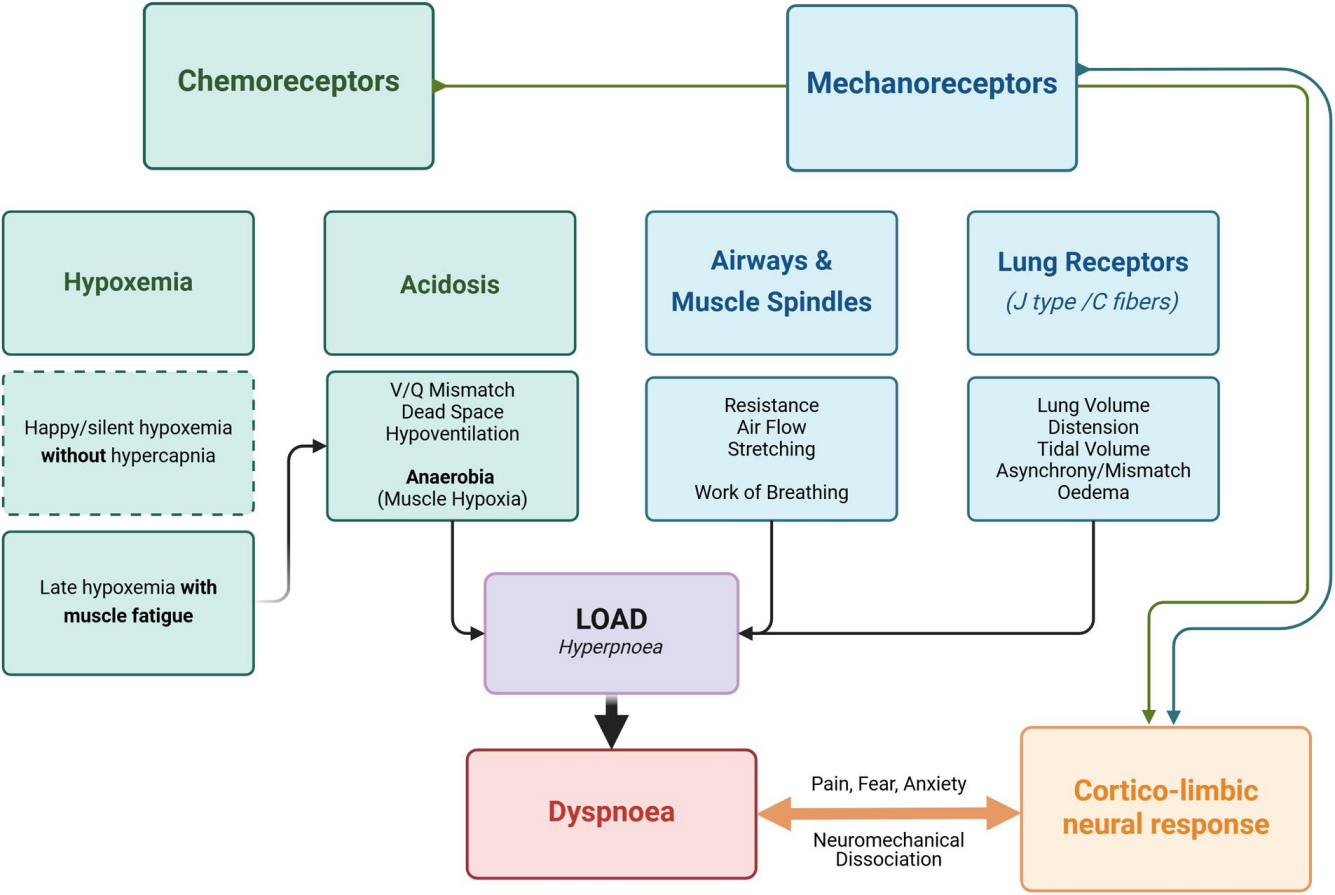
Principal known mechanisms generating dyspnoea in the respiratory centers. Created in BioRender. Romanet, C. (2026) https://BioRender.com/sh49m9w.

Dyspnoea is a familiar symptom to various health professionals, including pulmonologists, cardiologists, intensive care physicians and physiotherapists (1). Its onset is always an emergency and its persistence generates a major anxiety, often comparable to the fear of dying (2). Like pain, dyspnoea is multifactorial and its mechanisms are still not fully elucidated. Dyspnoea, as other archaic vital functions, is felt in the respiratory center of our brainstem, in the medulla (3). The onset of dyspnoea may be initiated by various intricate circuits (Figure 1) involving chemoreceptors (mostly sensitive to the pH and CO_2_) (4, 5); and mechanoreceptors (sensitive to the work of breathing and the mechanical properties of the lung) (6–8) which inform the individual on the actual load of the respiratory system. The dyspneic response is furthermore deeply linked with the cortico-limbic circuit, controlling our behaviors and emotions (fear, anxiety and even kinesiophobia) which can sustain dyspnoea or even initiate it *de novo* without organic stimuli (9–11). As such, the limbic response possesses the capacity to modulate (positively or negatively) this sensation of suffocation.

In the context of chronic diseases, the respiratory system may be under pressure from two main organic factors, eventually producing dyspnoea. First, the respiratory muscles may be incapable of functioning at higher levels during activities of varying intensity and in the worst case scenario, they may even be under strain during periods of rest. Second, the capacity of the lung parenchyma to efficiently provide gas exchange (intake of O_2_, release of CO_2_), as required by the cardiovascular system, may be impaired. The two phenomena are inherently interconnected, exerting a constant influence on one another. Finally, in some cases such as Chronic Hyperventilation Syndrome, the inadequate and regular activation of the limbic response may result in breathlessness without clear impairment of the former two. As a result, chronic breathlessness is a symptom that imposes limitations on exertion and, by extension, autonomy. Moreover, it has been demonstrated as a significant determinant of decline in health-related quality of life (HRQOL) (1). As well as having consequences for HRQOL, dyspnoea may in turn result in the worsening of respiratory diseases by the provoked sedentary lifestyle and the depression that is often associated with it, creating a vicious cycle (12).

While in acute cases the primary treatment for dyspnoea is to address the underlying condition, chronic dyspnoea which accompanies evolutive conditions, requires long-term treatments, the objective of which is to enhance exercise tolerance by increasing physical capacity, thereby alleviating dyspnoea. In order to achieve this challenging goal, pulmonary rehabilitation (PR) is the cornerstone of these treatments, and the most well described, studied and validated (13). PR are structured programs delivered by a multidisciplinary team. Beside education (14, 15) and self-management support (disease knowledge, behaviour change) (16, 17), assessment of the individual (18), tailoring to needs (physical, psychosocial), one physical treatment, exercise training is intended to improve both exercise capacity and dyspnoea (19–26). While quite studied and validated for chronic obstructive pulmonary disease (COPD) and chronic heart failure (CHF), accumulating evidence suggests that exercise training is beneficial in a broader range of conditions, including Post-Acute Covid syndrome (PACS) (27). Additionally, while exercise training in PR is highly protocolised, new modalities, contemporary to the COVID-19 pandemic have been put forward to optimise therapeutic efficiency or adhesion.

This state-of-the-art narrative review aims to provide a readable practical synthesis to help those learning about exercise training in pulmonary rehabilitation as well as those treating the individuals affected by chronic dyspnoea, whether in the context of a respiratory disease or not. The review focuses on the different uses of exercise training, from the historical to the emerging, the pathophysiological mechanisms by which it causes changes in the body, and future research directions.

## Methods

This narrative review was initiated on the basis of several meta-analyses, namely Khalafi et al., Oliveira et al., and Martinez-Pozas et al. (28–30). The COVID-19 pandemic in 2020 had indeed allowed for new evidence to emerge on rehabilitation modalities (telerehabilitation, virtual-reality, intensity of training) and indications (inflammation, PACS, obesity) for the treatment of breathlessness outside of the traditional scopes of PR. We thus chose to retrieve articles investigating the specific effects of exercise training on dyspnoea in the context of chronic diseases.

Studies were identified by a search in PubMed/MEDLINE (National Library of Medicine) and Cochrane databases, as well as clinicaltrials.gov for future directions, from January 2020 until April 2025, in English, by two independent authors (CR & JW). The final selection of articles was made by the three authors, as a function of their relevance to the addressed question. We included all relevant studies on design and questions raised. Ongoing research was also integrated. An emphasis was placed on meta-analyses regarding effectiveness of interventions. Yet, only articles which had focused on exercise training's effects (exercise training, whether implemented in PR or not, was determined by reading the methods) on patient-oriented dyspnoea outcomes (dyspnoea scales, functional measures, dyspnoea health-related quality of life scales) were kept for final analysis. Our team's perspective and analysis on the subject was influenced by our specific approach to dyspnoea, both acute in critical care in the context of sepsis, sub-acute in post-ICU consultations, and chronic in our outpatient PR activity.

Additional articles, cited in those already selected, were also reviewed and included if they added new information regarding the review. In this review, the terms *physiotherapy*, *rehabilitation*, *pulmonary rehabilitation*, and *exercise training* are used with their specific meanings to avoid conceptual overlap (1, 26). *Physiotherapy* refers to the healthcare profession providing a range of rehabilitative interventions aimed at restoring or maintaining physical function. *Rehabilitation* denotes a comprehensive, multidisciplinary process designed to optimize functional capacity and HRQOL in individuals with health conditions or disability. *Pulmonary rehabilitation* is a specific, evidence-based application of these principles to people with chronic respiratory diseases, integrating *exercise training*, education, and behavioural support. Keywords included “Pulmonary Rehabilitation”, “Training”, “Rehabilitation”, “Exercise Training”, “Physiotherapy”, “Dyspnoea”, “Breathlessness”, “Chronic”, “Intensity”, “Respiratory”.

The decision to conclude the research was made by the investigators collectively when the saturation of data was deemed achieved, i.e., when no further data emerged from the review of additional articles.

## Results

### Exercise training in pulmonary rehabilitation: established indications

The significant rise in chronic respiratory diseases over the XX^th^ century, especially COPD, has created a demand for specialised interventions aimed at slowing disease progression and alleviating breathlessness. PR was elaborated in this context and is nowadays supported by clinical trials and meta-analyses (13), establishing it as a Grade-A evidence-based intervention to improve exercise capacity, dyspnoea, and HRQOL, as reflected in international guidelines (31, 32).

Within PR, exercise training refers to the progressive and individually prescribed physical activity that constitutes the core therapeutic component of the programme, as opposed to the educational, behavioural and self-management elements that accompany it (1). It is the only component for which the evidence of benefit on exercise capacity, dyspnoea and HRQOL is consensual (26), and it is consistently identified as the main driver of clinical improvement. This was recently confirmed by the largest component network meta-analysis of PR to date, which pooled 337 randomised controlled trials and nearly 19,000 people with COPD: physiotherapist supervised, prescribed aerobic exercise training emerged as the minimum effective intervention, with in-person supervision adding a moderate increment to all three core outcomes, whereas commonly added components such as structured education, strength training and nutritional support showed little or no independent effect (24). Moreover, exercise training appeared most effective when prescribed at the highest intensity tolerated by the individual, and programme duration had no measurable influence on outcomes (24). Exercise training should therefore be understood not as a generic recommendation to “be active”, but as a supervised, intensity-targeted prescription, framed by an individual assessment of exercise capacity and tolerance (1, 13).

While PR is mostly established for dyspnoea management in COPD and CHF, its efficacy has been studied in several other respiratory conditions. In interstitial lung diseases (ILD), notably idiopathic pulmonary fibrosis for instance, exercise training can be implemented safely despite fixed restrictive ventilatory lung alteration limiting exercise tolerance, and is associated with meaningful improvements in functional capacity (6MWT: +40m, 95% CI 32.7 to 47.4), dyspnoea (standardised mean difference SMD −0.36, 95% CI −0.58 to −0.14) and HRQOL (St George's Respiratory Questionnaire SGRQ −9.29 95% CI −11.06 to −7.52) (33–35). In these studies, dyspnoea was predominantly assessed using the Modified Medical Research Council (mMRC) scale, the Borg scale during exercise, or disease-specific instruments such as the Chronic Respiratory Disease Questionnaire dyspnoea subscale or the SGRQ. Similar effects on exercise capacity (6MWT +34 m, 95% CI 18 to 51) and HRQOL [short-form health survey 36 mental health, 7.3 (± 2.5), *P* = 0.004] were also reported in pulmonary arterial hypertension, although dyspnoea was seldom evaluated in these trials (36–41).

### Exercise training in pulmonary rehabilitation: emerging indications

#### Post-Acute COVID-19 syndrome

The COVID-19 pandemic has revealed a new indication for exercise training, namely PACS, commonly known as “long-COVID” and defined by the World Health Organization as “the persistence or development of new symptoms three months after the initial SARS-CoV-2 infection, lasting for at least two months and not explained by an alternative diagnosis” (42). Indeed, shortly after the initial pandemic wave, this condition began to affect a substantial proportion of people (up to 400 million individuals)including those who had only experienced mild forms (43). Initially, up to 20% of those hospitalised in intensive care units (ICU) subsequently exhibited persistent symptoms (fatigue, dyspnoea, memory loss etc.), despite undergoing initial rehabilitation (2, 44, 45). Of note, the clinical manifestations of PACS have since then varied significantly in terms of both the presence and intensity of symptoms, and no clear correlation has been established between initial infection severity (ranging from the asymptomatic state to the acute respiratory distress syndrome) and the development of PACS (44, 46). Furthermore, a wide array of symptoms such as fatigue, muscle weakness and anxiety has been observed in up to 50% of individuals with PACS. Of those, dyspnoea has been identified as one of the most prevalent after fatigue (32, 47).

While observational studies have documented persistent impairment of diffusing capacity for carbon monoxide (DLCO) and pulmonary function indices (forced expiratory volume, vital capacity) in the months following acute infection, regardless of initial severity (48–51), many people experienced persistent dyspnoea and fatigue despite normal pulmonary function tests. This dissociation between functional respiratory parameters and symptom burden has been partly attributed to dysautonomia, postural orthostatic tachycardia syndrome (POTS), and small fibre neuropathy, which may substantially impair exercise tolerance independently of pulmonary or musculoskeletal limitations and require specific consideration prior to engaging patients in rehabilitation (32, 44).

Fatigue, in the context of SARS-CoV-2, should not be overlooked, as it is the most frequently reported symptom in PACS and has generated a substantial body of research on its origins and association with dyspnoea, muscle weakness, depression, weight etc (52–54). Expectably, individuals affected by fatigue often show significantly reduced physical capacity and HRQOL. The relationship with dyspnoea remains uncertain, although dyspnoea would appear to be independently associated with a greater burden of symptoms and poorer HRQOL (55). Some authors recently hypothesised the existence of at least two distinct dyspnoea-related phenotypes: those with pronounced fatigue, characterised by normal lung function where dyspnoea is secondary to fatigue; and those characterised by pulmonary function abnormalities where intrinsic pulmonary defects could result in dyspnoea (55). While both phenotypes are expected to benefit from rehabilitation, the determination of the precise intensity to be applied to each appears to be a key element in the research.

During initial period of pandemics, faced with a surge of people with PACS and limited evidence on its natural history, learned societies initially emphasised the critical importance of physiotherapy while leaving the decision on rehabilitation's method and intensity to the discretion of the treating therapist (32). These interventions were diverse in nature, ranging from passive treatments (56), therapeutic education (16), breathing exercises (57–63), low-to-moderate intensity tele-rehabilitation (50, 64–69) and up to exercise training programmes (19–22, 70–75). As it is often the case in rehabilitation studies, the marked heterogeneity of these interventions, combined with frequent absence of appropriate control groups, has hindered the formulation of evidence-based recommendations for physiotherapy in PACS (76). However, recent randomised controlled trials and meta-analyses have demonstrated significant and homogeneous improvements in symptom burden with high intensity exercise training alone (29, 30, 77, 78). For instance, one study reported a statistically significant and clinically meaningful reduction in the three dimensions of dyspnoea [Multidimensional Dyspnoea Profile: −18.61, 95% CI (−27.78 to −9.44), mMRC −0.76, 95% CI (−1.21 to −0.30)] following an outpatient exercise training programme in individuals with PACS (19). At the meta-analytic level, these results were confirmed with significant improvements in exercise capacity [6MWT: +60.56, 95% CI (40.75 to 80.36)], fatigue [Fatigue Severity Scale: −0.90, 95% CI (−1.49 to −0.31)] and dyspnoea [SMD −0.63, 95% CI (−1.03 to −0.24)] across included studies (29, 30, 77, 79). Targeting submaximal exhaustion and dyspnoea during exercise training, appears to have played a significant part in allowing for both a strong and reproducible effect to be discovered, supporting its key role in PACS rehabilitation.

#### Obesity, systemic inflammation and neurological conditions

Obesity, now recognised as a major chronic inflammatory disease associated with malnutrition, impaired exercise capacity, exertional dyspnoea and reduced HRQOL (80), has been investigated as a potential indication for PR (81). It is important however to acknowledge that obesity is seldom studied isolated within rehabilitation literature. It is instead mostly examined as a comorbidity with concurrent cardiovascular, respiratory or vascular diseases, particularly COPD and asthma which limits the generalisability of available evidence to people with obesity-related dyspnoea in the absence of documented lung disease. Nevertheless, several studies have highlighted dyspnoea improvements after exercise training alone (82–85), and notably, following comprehensive PR programs including non-invasive ventilation, nutrition and psychological follow up (86).

Of note, concerns that high-intensity exercise training might exacerbate systemic inflammation have not been supported by subsequent evidence. On the contrary, recent meta-analyses have demonstrated substantial reductions in circulating inflammatory markers following exercise training in people with obesity (87, 88), type 2 diabetes (89) and cancer (90). Moreover, these anti-inflammatory effects appear greatest in individuals with pre-existing chronic low-grade inflammation, including older adults with comorbid chronic disease, compared to healthy controls, suggesting a potentially amplified benefit of exercise training in the populations most affected by chronic dyspnoea (28).

Exercise training has also been evaluated in neurological conditions where pulmonary function is generally preserved but where motor deficits, reduced thoracic excursion, and respiratory muscles weakness may contribute to chronic dyspnoea. After stroke, exercise training has been shown to be safe and resulted in significant and clinically important changes in VO2peak (+4.13 95% CI 2.44 to 5.82) and 6MWT (+88.87 95% CI 29.08 to 148.67) (91–98). In Parkinson's disease, while studies on exercise training are less common, the results are no less encouraging and share the same trend with significant improvements in VO2peak, 6MWT as well as perceived fatigue (Parkinson's Disease Fatigue Scale), symptoms (Uniﬁed Parkinson's Disease Rating Scale) and HRQOL (39-item Parkinson's Disease Questionnaire (99–102). Finally, fewer studies have evaluated exercise training in the context of multiple sclerosis. A systematic review of 19 studies in people with severe mobility disability (Expanded Disability Status Scale EDSS ≥ 6.0) found limited but encouraging effects of exercise training on disability (EDSS), physical fitness (VO2peak, muscle strength), physical function (6MWT, Berg Balance Scale, TImed Up and Go), fatigue and HRQOL (103). However limitations exist: first, while exercise training was feasible and safe, it yielded little to no statistically significant effects. Second, expensive adapted modalities designed for limited mobility (body-weight-support treadmill, recumbent stepping and electrical-stimulation cycling) were used, limiting external validity (104). Third, the studies were small, methodologically heterogeneous, and rarely included controlled designs, so the optimal prescription and the durability of these effects remain undefined.

As a whole, while those trials highlight feasibility in these neurologic disorders and show promising results towards physical function and HRQOL, dyspnoea itself has rarely been the primary outcome, and limited available data should therefore be interpreted with caution in terms of direct applicability to dyspnoea management.

#### The ICU and post-ICU context

The role of exercise training in the ICU and post-ICU settings is a more intricate matter. ICU-acquired weakness, encompassing critical illness polyneuropathy and critical illness myopathy, affects an estimated 40%–60% of those receiving prolonged mechanical ventilation (105). In this context, the following factors have been identified as major contributors to muscle wasting and peripheral nerve injury, all of which may have lasting effects on an individual's autonomy following discharge: bed rest, systemic inflammation, sepsis, sedation and neuromuscular blockade. Beyond peripheral skeletal muscle dysfunction, diaphragmatic atrophy secondary to mechanical ventilation constitutes a recognised and clinically significant contributor to dyspnoea and weaning failure in this population (106–108). During ICU stay, early rehabilitation and exercise training strategies have been extensively investigated; however, to date, current evidence remains inconclusive, with several large randomised trials failing to demonstrate clear and significant effects on function (109). While early active mobilisation has shown benefit over late or no mobilisation in some studies (110), the optimal rehabilitation dose remains undefined, and no consistent effect on ICU length of stay, ventilator-free days or mortality has been established (111, 112). These negative results likely reflect, at least in part, the inherent difficulty of delivering high-intensity exercise training to critically ill, haemodynamically unstable patients, as well as the marked clinical heterogeneity of this specific population. This is why several authors have chosen to investigate exercise training after discharge, in the post-intensive care syndrome (PICS).

Post-ICU rehabilitation is itself complicated by the PICS which shares similarities with PACS. Characterised by persistent physical, cognitive and psychiatric sequelae and an associated frailty phenotype, PICS combines peripheral amyotrophy, respiratory muscle dysfunction, frailty, and neuropsychological sequelae (105). These overlapping deficits call for an individualised, multidisciplinary approach to rehabilitation. Nevertheless, despite encouraging results in a few randomised controlled trials, post-ICU exercise training has also failed to show significant improvements on functional capacity, dyspnoea or HRQOL in a Cochrane recent meta-analysis (113), a result probably attributable to substantial between-study heterogeneity. Updated meta-analyses including more recent studies, including those conducted in PACS (77, 114, 115) and those ongoing (NCT05218083) are needed to clarify the role of post-ICU exercise training in this population.

### Mechanisms underlying the effect of exercise training on dyspnoea

#### Deconditioning, sarcopenia and chronic dyspnoea

Chronic dyspnoea in people with respiratory disease arises from the association of multiple pathophysiological mechanisms that extend well beyond peripheral muscle deconditioning alone (116). Recognising this multifactorial basis is essential to avoid reducing the pathophysiology of chronic dyspnoea to deconditioning alone, and to ensure that exercise training is implemented within a comprehensive, individually tailored clinical assessment.

Dynamic hyperinflation deserves particular attention, as it is one of the main mechanical determinants of exertional dyspnoea in COPD (117). Expiratory flow limitation leads to progressive air-trapping during exercise through the increase of respiratory rate and tidal volume, quickly raising end-expiratory lung volume and reducing inspiratory capacity. Tidal volume is thus displaced towards the upper, less compliant portion of the pressure-volume curve, increasing the work load on the inspiratory muscles while placing the diaphragm at a mechanical disadvantage (shortened). The resulting constraint on lung expansion, despite a strong neural drive to breathe, produces a neuromechanical dissociation that correlates with the intensity of dyspnoea and the feeling of unsatisfied inspiration (116–118) (Figure 1). This phenomenon may partly explain the often described discordance between resting pulmonary function and exertional symptom burden. In restrictive disorders such as ILD, by contrast, ventilatory limitation arises chiefly from reduced lung volumes and a high ventilatory demand relative to a fixed maximal ventilation, again limiting exercise capacity (119, 120). Cardiovascular dysfunction, including reduced cardiac output, chronotropic incompetence, and pulmonary vascular remodelling in conditions such as pulmonary hypertension, constitutes an additional contributor to exertional dyspnoea (36–40). Nevertheless, the importance of peripheral muscle dysfunction in chronic dyspnoea has been quite studied (116). Early discoveries of skeletal muscle atrophy in those confined to prolonged bed rest (121, 122) as well as the switch from type 1 to type 2 muscle fibres in those with chronic respiratory disorders (123) emphasised the importance of muscle training in maintaining musculoskeletal integrity. This fibre-type shift is a natural phenomenon of remodelling in response to the mechanical stress and strain to which it is subjected, largely opposite to the adaptations sought through exercise training. In the limb muscles of people with COPD, the proportion of fatigue-resistant slow oxidative type I fibres is reduced in favour of glycolytic, fast type II fibres, alongside a decline in oxidative enzyme activity and capillary density (12, 116). These changes promote earlier reliance on anaerobic metabolism and accelerated lactate accumulation at low workloads, raising the ventilatory demand for a given effort and feeding back into the hyperinflation and ventilatory mechanisms described above (Figure 1). Their severity broadly parallels disease progression, and they are at least partially reversible with strength and endurance training, providing a direct rationale for prioritising aerobic exercise training in this population (12, 23, 25, 124).

**Figure 2:**
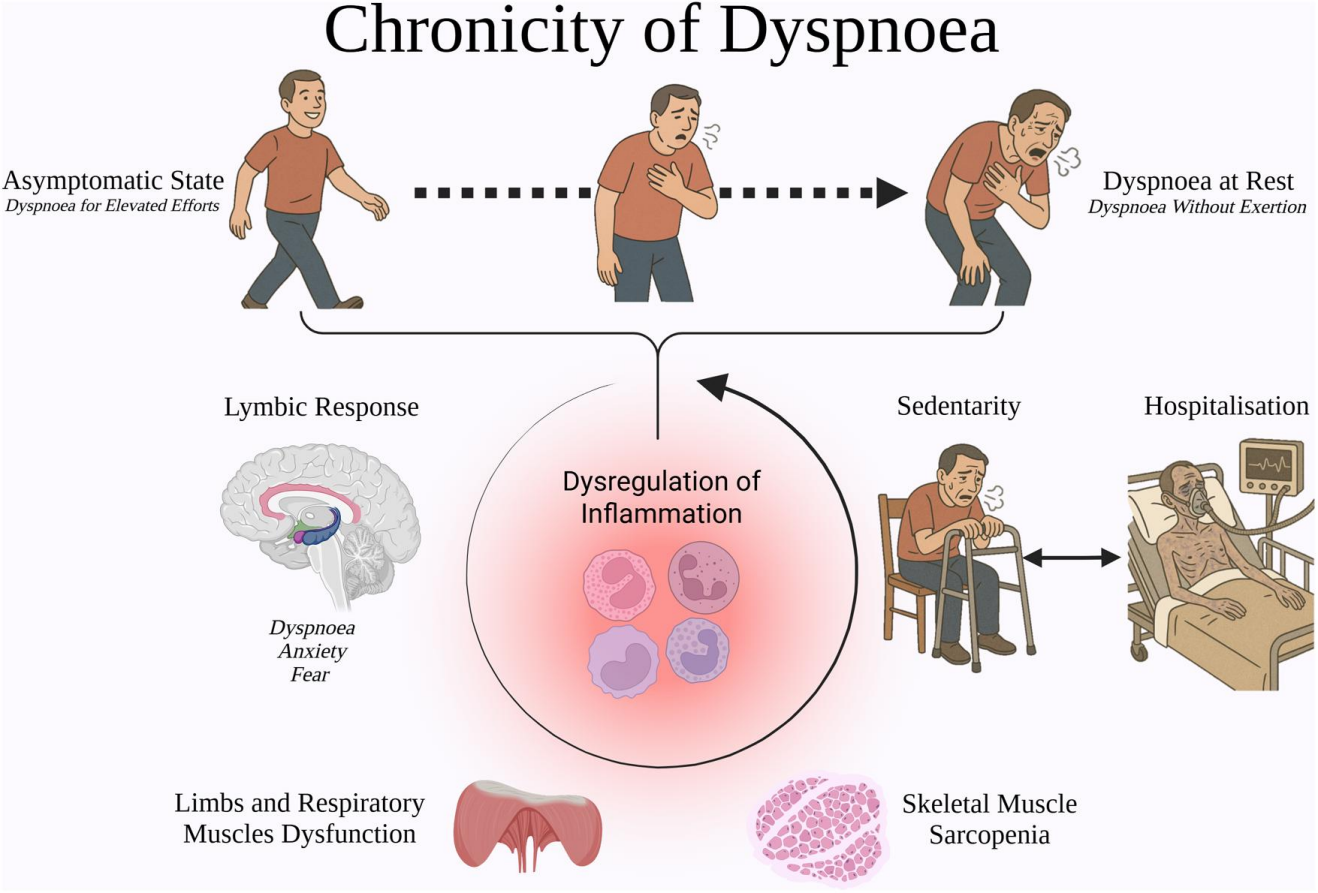
Visual representation of the mechanisms involved in the process of deconditioning, which can result in the chronicity of dyspnoea. Created in BioRender. Romanet, C. (2026) https://BioRender.com/jnwcooe.

In the absence of adequate mechanical stimulation, progressive muscle atrophy occurs through several identified pathways (125–131), while excessive loading may injure cytoskeletal components (25, 106–108, 132–134). Both underuse and overuse, compounded by systemic inflammation and oxidative stress, may therefore contribute to further deconditioning and exacerbate both resting and exertional dyspnoea (Figure 2). While limb muscle dysfunction has been identified and may be a pivotal component in limitation of daily activities, underuse or overuse are likely pieces of the puzzle rather than the sole explanation for muscle impairment in this population, given the additional roles of inflammation, hypoxaemia, nutritional deficits (12, 135, 136). However, in addition to treating these latter elements, strengthening dysfunctional muscles to improve aerobic capacity remains a central evidenced-based objective of rehabilitation in chronic respiratory diseases (12, 124).

In PACS, similar mechanisms seem to be involved in the development of dyspnoea and reduced exercise tolerance. First, in the acute stage, the viral infection has been shown to induce inflammatory lesions within the lung parenchyma and disrupt other organs, such as skeletal muscles (137–139). Progressive respiratory muscle impairment, driven by the combination of systemic inflammation, hypoxia-related fatigue, and increased work of breathing, may ultimately result in diaphragmatic dysfunction, a recognised contributor to dyspnoea in this population (47, 140). Subsequent prolonged immobilisation during and after hospitalisation (141), creates favourable conditions for peripheral amyotrophy and a sustained inflammatory state (44, 141–143), both of which are associated with reduction in exercise capacity, functional autonomy, and HRQOL (50, 57, 58, 144, 145). Importantly, as it is the case in COPD, these phenomena are intertwined with other mechanisms previously described (dysautonomia, hyperventilation, fatigue etc.) which explain both the variability of clinical presentations and the heterogeneity of responses to rehabilitation in this population.

Taken together, these observations show that the dyspnoea accompanying chronic disease cannot be reduced to a single cause. The relative weight of each mechanism varies between individuals and diseases, but their common denominator is a downward spiral in which symptoms restrict activity, activity restriction worsens deconditioning, and deconditioning amplifies symptoms. It is precisely this self-perpetuating vicious cycle that exercise training is aimed at interrupting (12).

#### The role of exercise intensity: neurochemical, aerobic and structural mechanisms

**Figure 3:**
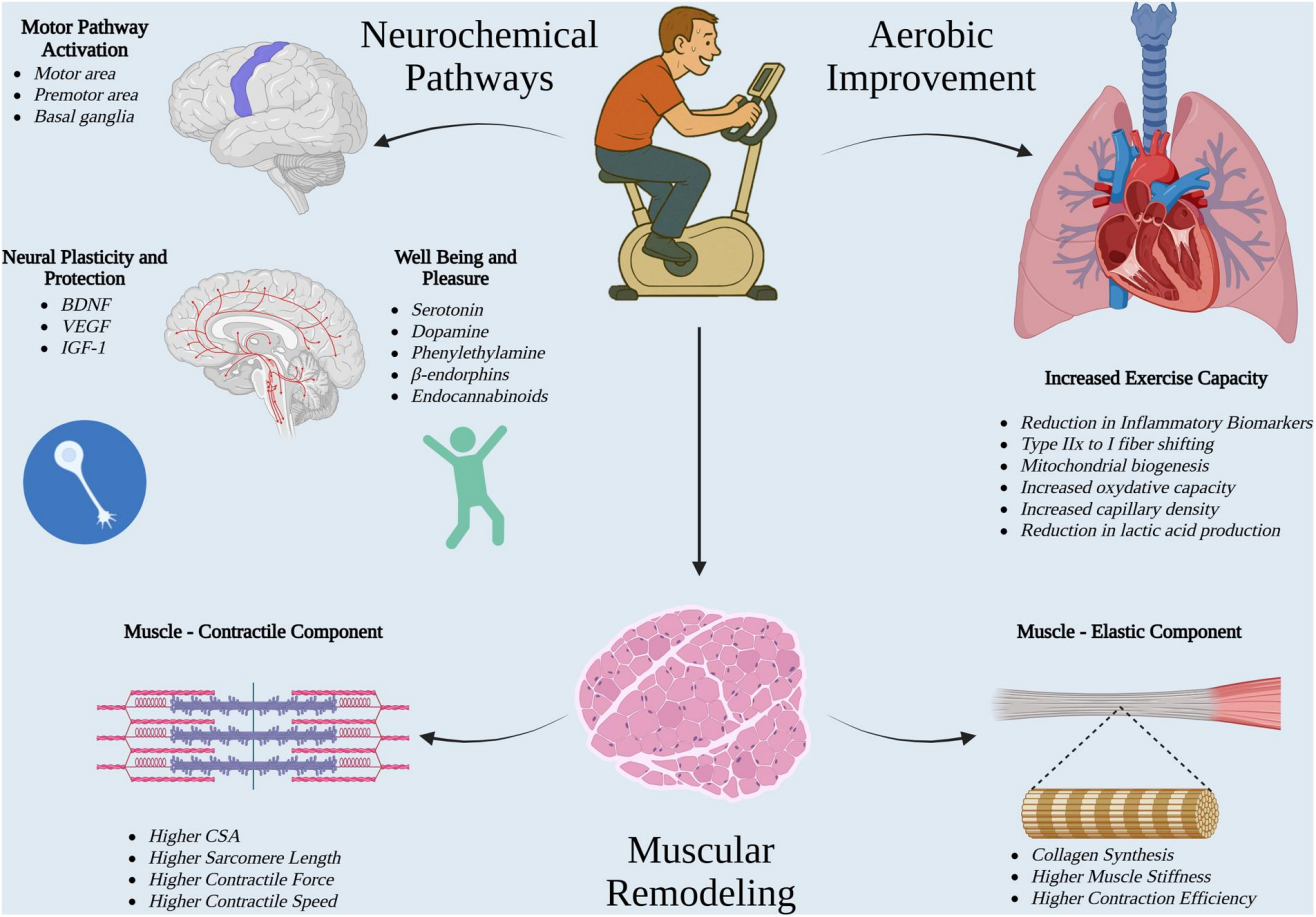
Key effects of exercise training in the context of chronic dyspnoea. (CSA, Cross Section Area; VEGF, Vascular Endothelial Growth Factor; BDNF, Brain Derived Neurotrophic Factor; IGF1, Insulin-like Growth Factor 1). Created in BioRender. Romanet, C. (2026) https://BioRender.com/n7c3nky.

If exercise training can act on such a diversity of mechanisms, the question that follows is why intensity of exercise, rather than exercise *per se*, appears to determine its effectiveness. Indeed, across populations, the studies that produced the most consistent and reproducible improvements in dyspnoea, exercise capacity and HRQOL appear to be those that reached a sufficient training intensity. Understanding why requires looking into the adaptations intensity elicits at three interacting levels: neurochemical, aerobic and structural (Figure 3).

#### Neurochemical component

Dyspnoea is by itself paradoxical in rehabilitation: it is both the primary target of rehabilitation and a significant limiting factor to physical activity due to its unpleasant nature, its association with fear of dying and anxiety (146). Lack of physical activity, or a sedentary lifestyle, is widely acknowledged as one of the most modifiable risk factors contributing to numerous diseases, including neurodegenerative diseases such as Alzheimer's and Parkinson's disease (147–150). Yet exercise training may offer a distinctive form of counterbalance by stimulating specific neurochemical responses. Exercise training stimulates the secretion of neurotransmitters such as dopamine and serotonin, which play essential roles in regulating fatigue, pleasure, and cognitive function (151). Additionally, the release of phenethylamine, beta-endorphins, and endocannabinoids enables a sense of well-being after exercise (commonly referred to as the “runner's high”) (151–155). This response is particularly pronounced in exercises exceeding 60% VO₂^Peak^, and has been linked to reductions in anxiety and stress (151–154, 156), both of which are known to exacerbate dyspnoea (2, 157). Beyond acute effects, these neurochemical responses play a pivotal role in reinforcing positive behaviour and favouring long-term adherence to rehabilitation programs (156). Regular high-intensity exercises have also been associated with neuroplastic adaptations (155) that enhance motivation and therapeutic adherence, making exercise training not only a physically beneficial intervention but also a psychologically empowering experience (151).

#### Aerobic component

In addition to these psychological and neurochemical benefits, exercise training is well-documented for its physiological impact on aerobic capacity (26). However, achieving these benefits requires that muscular fatigue and dyspnoea be actively pursued during training, implying that exercise intensity is sufficient to elicit meaningful physiological improvements (124, 158). While both high intensity interval training (HIIT) and moderate-intensity continuous training (MICT) can produce clinically significant improvements in exercise tolerance, peripheral muscle function, and overall respiratory efficiency (144, 159–162) the choice of training modality should however be individualised based on the subjects’ preference and tolerance (163). Physiological adaptations common to both modalities include mitochondrial biogenesis, enhanced muscle oxidative capacity, and increased capillary density, improving aerobic performance. In addition, in people without dynamic hyperinflation, the higher tidal volumes achieved during intense exercise may activate pulmonary slowly adapting stretch receptors and elicit the Hering-Breuer reflex, transiently attenuating air hunger and facilitating further exertion (164). These adaptations shift the dyspnoea threshold towards higher levels of physical activity by reducing ventilatory demand and lactate accumulation at any given workload.

#### Structural component

Finally, beyond these neurochemical and aerobic effects, exercise intensity plays a crucial role in structural adaptations of the muscle-tendon unit. High-intensity exercise training induces significant histologic remodeling of the muscle-tendon unit, enhancing both neuromuscular performance and structural integrity (162, 165). The repeated bouts of maximal or near-maximal efforts promote muscle hypertrophy, increasing cross-sectional area and contractile force, even in older individuals (166). Biomechanically, the remodelling of muscle-tendon units is mostly stimulated during eccentric contraction, whereby contracted muscles are forced to stretch (167). The eccentric components inherent to numerous high-intensity exercise modalities, such as plyometrics and controlled deceleration phases, impose greater mechanical stress on the tendons, stimulating collagen synthesis and structural strengthening (168, 169). Consequent increases in tendon stiffness optimise force transmission and reduce energy dissipation during repetitive dynamic tasks, thereby lowering the metabolic cost of locomotion (170–172). However, this process takes time (between 12 and 16 weeks of loading) and excessive training volume or inadequate recovery may predispose muscles and tendons to overuse injuries (168). This is why, in order to ensure optimal gains while minimising injuries, the presence of a physiotherapist is essential to carefully monitor and adjust the loading and recovery process for each individual (167).

In PR, exercise training is actually predominantly delivered via conventional concentric cycle ergometry, which limits the scope of eccentric stimulation. Emerging evidence supports the feasibility and safety of eccentric cycle ergometers in patients with chronic cardiac and respiratory conditions (173–177) with demonstrated superiority over concentric ergometry for muscle mass and strength gains in COPD (178–180). A potential reduction in perceived dyspnoea (64.4 ± 29.6% lower dyspnea, *p* < 0.001, in Inostroza et al., 28.2 ± 31.7% lower dyspnoea, *p* < 0.05, in Bourbeau et al.) has been reported during eccentric compared to concentric cycling in exercise training (178, 179). While the subject's muscle mass and strength had increased, the observed modulation of dyspnoea could potentially be attributed not to the increase in contractile muscle fibres, but rather to the increase in tendinous structures (tendon, aponeurosis, titin), which increase the force produced during contraction (storage of elastic energy) thus improving muscular efficiency at reduced metabolic cost (169, 179). It should be noted, however, that these mechanistic explanations remain partly hypothetical given the limited number of available trials and their methodological heterogeneity. These hypotheses, while physiologically plausible, require confirmation from adequately powered clinical studies, such as the ongoing multicentre trial (NCT06168266) which may provide further insight into the therapeutic potential of eccentric training in modulating dyspnea (181).

While higher intensities of exercise training are indeed associated with superior physiological adaptations, it must be clear that they are neither universally achievable nor universally safe. Those with advanced COPD, severe pulmonary hypertension, significant exercise-induced desaturation, or marked restrictive profile may not tolerate sustained high-intensity continuous exercise, and interval-based or adapted modalities should be preferred in these populations (163). Hence, the main objective is to reach the highest individually tolerable intensity, considerably exceeding the loads encountered during activities of daily living, rather than to apply a uniform prescription. The importance of individually adapted exercise training intensity in PR is well documented in COPD (163) and CHF (182), and has more recently been confirmed in PACS by randomised controlled trials and meta-analyses (29, 77, 78).

### Exercise training: in practice

#### Generalities

The expanding scope of exercise training across diverse disease states makes it necessary to provide practical guidance on subjects’ selection, assessment, monitoring and implementation (183). Exercise training must be progressive and tailored to each individual. It is most commonly delivered on a cycle ergometer or a treadmill, either as continuous training or as interval training. Intensity of training is operationally defined by objective parameters including the percentage of peak oxygen uptake (VO_2_^peak^) or maximum heart rate, as well as subjective measures such as the rating of perceived exertion (Borg scale) (163, 182).

Contraindications must be respected at every stage. Absolute contraindications include, but are not limited to, unstable angina or arrhythmia, unstable bone fracture, acute infectious disease, acute cor pulmonale, and unstable psychiatric conditions or dementia (183). In PACS specifically, dysautonomia and POTS should be screened prior to initiation, as they may cause haemodynamic instability during exertion and require adapted protocols, including progressive upright positioning and a low-intensity warm-up before higher-intensity training (27).

#### Baseline assessment

As outlined earlier, exercise training should only be initiated after a careful individual assessment of exercise capacity and tolerance, both to quantify functional impairment, detect exercise-induced desaturation (SpO₂ < 88%–90%), and guide individualised intensity prescription (1, 184, 185). Cardiopulmonary exercise testing (CPET) is the reference method, allowing physiological variables such as VO₂^peak^, heart rate and workload (in watts) to be measured at different thresholds; however, it requires specific, costly equipment and trained medical staff. In practice, field tests are most frequently used, principally the 6MWT and the incremental shuttle walk test. Whichever test is used, its purpose is to confirm the safety of exercise training and to validate the prescription in terms of intensity, duration, and the choice between continuous and interval training. Other easily implemented functional tools exist, requiring little equipment (e.g., the 1-minute sit-to-stand or constant and incremental step tests), but they remain poorly described outside COPD and their use for rehabilitation prescription is not currently recommended (18).

#### Intensity and modality

Once exercise capacity is established, training intensity is prescribed according to the difficulty targeted by the physiotherapist (Table 1) (24, 186):

- low intensity, inducing little or no dyspnoea and only a minor increase in heart rate, should be set below 50% of the peak work rate on a cycloergometer, or below 70% of the 6MWT walking speed;
- moderate intensity, inducing moderate symptoms, should be set between 50%–70% of the peak work rate on a cycloergometer, or between 70%–80% of the 6MWT walking speed;
- high intensity, inducing dyspnoea above 4/10 on a numerical scale and more difficult to sustain over prolonged periods. In this context, training will target a work threshold above 80% of the peak work rate, or a walking speed above 80% of the 6MWT.

**Table 1:** Exercise training in endurance across different populations, summary of recommendations. COPD, chronic obstructive pulmonary disease; ILD, interstitial lung disease; PACS, Post-acute COVID syndrome; Wpeak, Peak work rate; 6MWT, 6-minute walking test; IT, interval training.

Settings	COPD (161, 162, 186)	ILD (34, 35)	PACS (30, 79)	Obesity (81)	Neurologic disorders (93, 98)
**Total Duration program**	8–12 Weeks	8–12 Weeks	6–12 Weeks	8–12 Weeks	4–24 Weeks
**Frequency** **Endurance training Duration**	2 to 3 sessions/week				3 sessions/week
*Training duration for IT should be longer as it entails resting periods*	30 to 40 min	30 to 40 min	20 to 40 min	20 to 60 min	20 to 60 min
**Warm up**	≤ 5 min				
**Continuous training** ^a^	Moderate to high intensity with cycling at 50–80% Wpeak or walking at 70–80% of walking speed (6MWT).	Moderate to high intensity with cycling at 60–70% Wpeak or at 70–80% of walking speed (6MWT).	Low to moderate continuous intensity with cycling at 40–60% Wpeak.^b^	Moderate to high intensity with cycling at 50–70% of VO2peak or walking at 70–80% of walking speed (6MWT)	Moderate to high intensity with cycling at 50–75% of Wpeak or walking at 60–80% of heart rate reserve; with a perceived dyspnoea above 5/10
**Interval training** ^a^ *Should be set as follows:* *Series of training at target worload/rest or active recovery*	High intensity with cycling at >80% Wpeak, with several options for splitting: • 30s/30s • 20s/40s • 60/120s or 120/60s The longer the duration, the lesser the workload	Less described 30s/30s cycling at 100% Wpeak	Interval training is seldom described 30s/30s cycling at 80%–90% Wpeak	High intensity with cycling at 90 to 130% of Wpeak	High intensity with cycling at >95% Wpeak, with progression on the bouts’ duration: • 30s/60s • 45s/60s • 60s/60s

a whether opting for continuous or interval training, the initial sessions may be shorter before progressively increasing the duration with the sessions up to the target duration.

b recommendations are less precise, often based on symptoms expressed by the individua.

Sessions should start with a 5 min warm-up, at a lower intensity than the one targeted during training. The choice of modality follows from the targeted intensity. Low-to-moderate intensity is generally delivered as continuous training (LICT and MICT), whereas high-intensity training may be harder for some individuals to sustain continuously; interval training (HIIT) is then a suitable alternative, particularly where a constant work rate cannot be maintained because of either breathlessness or desaturation (161). When training is delivered on a treadmill, prescription is facilitated by the results of the 6MWT; on a cycle ergometer without a CPET, prescription is more difficult, and although formulae predicting VO₂^peak^ and work rate from the 6MWT exist, their precision remain limited. Beyond physiological considerations, adherence and tolerability are key determinants of long-term benefit, so the choice between HIIT and MICT should also account for individual preference and dyspnoea-related anxiety (11, 24, 186).

#### Monitoring during exercise

Because exercise training deliberately reproduces symptoms (dyspnoea, tachycardia, muscular fatigue) and places sustained demand on the cardiorespiratory system, monitoring during sessions is indispensable. Cardiovascular monitoring, including continuous heart rate, intermittent blood pressure measurement, and pulse oximetry, is recommended throughout each session, particularly during the initiation phase and in individuals with comorbid cardiac disease or significant ventilatory limitation (185). Supplemental oxygen should be provided during sessions in those with significant exertional desaturation. Physiotherapist supervision throughout the programme is essential, both to enable progressive and safe intensity titration and to ensure recognition of adverse events or exacerbations.

Telerehabilitation, put forward during the SARS-CoV-2 pandemic, may however also improve access and adherence in patients with mobility limitations or geographical barriers (187–193), provided that safety monitoring is maintained (24).

#### Heterogeneity of rehabilitation programmes

One of the principal limitations of the current literature is the marked heterogeneity of the protocols proposed, which complicates both their comparison and their translation into standardised practice. This heterogeneity concerns contents (intensity prescribed and the use of continuous versus interval training), but also the dose, namely session duration, frequency, and overall length. Frequencies of two to five sessions per week over eight to twelve weeks are commonly recommended, yet no consensus has been reached, and the available evidence does not allow a minimum effective duration to be defined. Notably, the recent component network meta-analysis by Ward et al. found that neither programme duration nor total number of sessions influenced the three core outcomes of exercise capacity, breathlessness and HRQOL (24), highlighting that intensity and supervision, rather than volume alone, appear to drive the benefit of exercise training. This persistent variability justifies the need for better-standardised and reported protocols in future trials.

#### Future direction

Despite its proven efficacy, exercise training remains underutilised inside and outside COPD management (187–193), necessitating broader advocacy for its integration into routine COPD care. Expanding its implementation to a broader range of respiratory and systemic conditions is both a logical and necessary step to improve individuals’ dyspnoea and by doing so, HRQOL and functional capacity. Future research should further investigate the importance of high-intensity training and eccentric contractions in PR and define its place into standard care for all persons experiencing dyspnoea. As the field of rehabilitation medicine advances, exercise training could be recognised as a potent intervention for respiratory health, rather than a niche therapy reserved for COPD.

#### Summary

Exercise training has been demonstrated to be an efficient modality of rehabilitation in alleviating dyspnoea in a wide range of conditions.

Exercise training should not be limited to COPD or CHF, and instead be proposed to people with chronic dyspnoea in non-respiratory conditions.

In exercise training, intensity is pivotal in achieving the effects on dyspnoea, exercise capacity and HRQOL, but must be individualised to tolerance and safety.

The potential for enhancement of exercise training through the incorporation of eccentric exercise training, a proven and efficient form of rehabilitation, remains to be explored.
